# Specific Immunosuppression with Inducible Foxp3-Transduced Polyclonal T cells

**DOI:** 10.1371/journal.pbio.0060276

**Published:** 2008-11-11

**Authors:** Kristian G Andersen, Tracey Butcher, Alexander G Betz

**Affiliations:** Medical Research Council, Laboratory of Molecular Biology, Cambridge, United Kingdom; National Jewish Medical and Research Center/Howard Hughes Medical Institute, United States of America

## Abstract

Forkhead box p3 (Foxp3)-expressing regulatory T cells are key mediators of peripheral tolerance suppressing undesirable immune responses. Ectopic expression of Foxp3 confers regulatory T cell phenotype to conventional T cells, lending itself to therapeutic use in the prevention of autoimmunity and transplant rejection. Here, we show that adoptive transfer of polyclonal, wild-type T cells transduced with an inducible form of Foxp3 (iFoxp3) can be used to suppress immune responses on demand. In contrast to Foxp3-transduced cells, iFoxp3-transduced cells home “correctly” into secondary lymphoid organs, where they expand and participate in immune responses. Upon induction of iFoxp3, the cells assume regulatory T cell phenotype and start to suppress the response they initially partook in without causing systemic immunosuppression. We used this approach to suppress collagen-induced arthritis, in which conventional Foxp3-transduced cells failed to show any effect. This provides us with a generally applicable strategy to specifically halt immune responses on demand without prior knowledge of the antigens involved.

## Author Summary

Autoimmune diseases come in many diverse forms—such as rheumatoid arthritis, type I diabetes, multiple sclerosis, and inflammatory bowel disease—yet all share the same underlying cause, the launch of a detrimental immune response. In healthy individuals, a specialized immune cell type called regulatory T cells prevents these undesirable immune responses. Here, we present a strategy to suppress undesirable immune responses using genetically modified proinflammatory T cells that participate in these inappropriate immune responses until they are activated with a drug. At this point, the genetic modification causes them to change their behavior to that of regulatory T cells. Using a mouse model, we demonstrate that this approach can be used to stop undesirable immune responses on demand with minimal intervention.

## Abstract 3

An approach to induce specific tolerance--an important application for the treatment of autoimmune diseases and transplant rejections--uses polyclonal T cells transduced with an inducible form of Foxp3.

## Introduction

Transplant rejection and autoimmune diseases ranging from rheumatoid arthritis, type I diabetes, and multiple sclerosis to inflammatory bowel disease—as diverse as they might appear—all have the same underlying problem: the launch of an undesirable immune response [1]. Equally similar are the current approaches to treat these conditions, which are generally based on drugs that lead to systemic immunosuppression [2]. Thus, the induction of specific tolerance is seen as the “Holy Grail” of therapeutic approaches [3].

The discovery that the immune system evolved regulatory T (T_R_) cells to stop undesirable immune responses, such as autoimmunity [4] and the rejection of the fetus [5–7], is of obvious therapeutic promise [8]. Indeed, T_R_ cells have already been shown to be capable of fulfilling such functions [9]. However, the translation of experimental findings into actual therapeutic approaches is hampered by a variety of problems. Under experimental conditions, antigen-specific tolerance can be achieved by using T_R_ cells from TCR-transgenic animals or by ex vivo expansion of antigen-specific T_R_ cells [9–11]. However, it is difficult to imagine how a TCR transgenic approach can be translated into a generally applicable therapy. The antigen-specific ex vivo expansion of T_R_ cells [9–11], or in vivo conversion of helper T cells (T_H_) into T_R_ cells [12], is more feasible, albeit still problematic. They not only rely on the knowledge of, or at least access to the antigens involved in the pathological immune response, but are also time consuming and complicated when applied in a therapeutic context [8,13].

There are also conceptual problems. The lack or malfunction of T_R_ cells is suspected to be at the root of many autoimmune diseases [14,15]. In these cases, it might be impossible to obtain and expand functional, antigen-specific T_R_ cells, as they may not exist in the host in the first place. In principle, this problem can be circumvented by the conversion of conventional T cells into T_R_ cells, either by TGF-ß–mediated induction [16–18] or ectopic expression of the lineage factor Forkhead box p3 (Foxp3) (NP_473380) [19–21]. However, without enriching antigen-specific “induced T_R_ cells,” this is likely to be of limited benefit and may lead to systemic immune-suppression [11,22–24]. A further problem with TGF-ß–induced T_R_ cells is that their phenotype seems to be unstable [25,26], although the presence of retinoic acid appears to stabilize the conversion [27,28].

Here, we present a strategy to suppress undesirable immune responses in an antigen-specific fashion without prior knowledge of the antigens involved. We accomplish this by adoptive transfer of a small number of polyclonal T_H_ cells transduced with a genetically engineered, inducible form of Foxp3 (iFoxp3). CD4^+^CD25^−^ cells transduced with iFoxp3 (T_H_::iFoxp3) initially retain their “proinflammatory” phenotype. They home “correctly” into the secondary lymphoid organs and partake in immune responses. Once the T_H_::iFoxp3 cells have expanded in an antigen-specific fashion, they can be converted to T_R_ cell phenotype on demand by inducing iFoxp3, thereby stopping the immune response they partook in.

## Results

### Failure of Polyclonal T_H_::Foxp3 Cells to Suppress Collagen-Induced Arthritis

**Figure 1:**
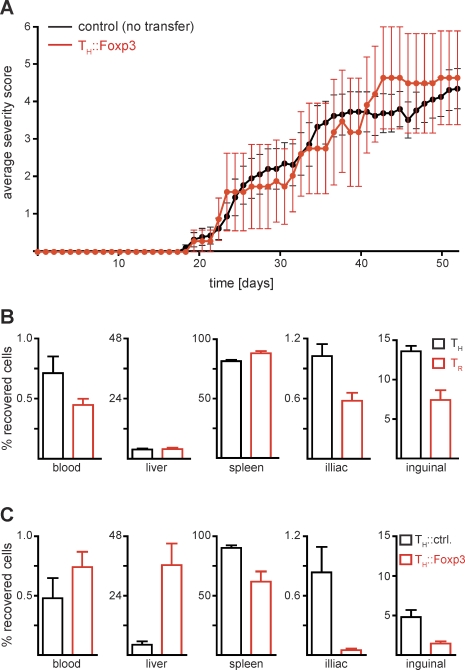
Polyclonal T_H_::Foxp3 Cells Fail to Suppress CIA and Exhibit Altered Homing Behavior (A) Arthritis was induced on day 0 by immunization with cII in CFA. Mice that did not receive any further treatment (black, *n* = 27) and mice that received 1 × 10^6^ T_H_::Foxp3 cells 1 d prior to immunization (red, *n* = 7) are shown. The average arthritis scores of all mice in the two groups are shown. (B and C) Comparison of the homing behavior of (B) CFSE-labeled T_H_ (black) and T_R_ (red) cells and (C) GFP-expressing T_H_::control (black) and T_H_::Foxp3 (red) cells. A total of 1 × 10^6^ cells were transferred into each mouse (T_H_, *n* = 3; T_R_, *n* = 3; control, *n* = 4; and Foxp3, *n* = 6), and the tissues were analyzed 48 h later by flow cytometry. The diagrams represent the percentage of cells in each tissue, calculated from the total number of cells recovered in all tissues together (1.2 × 10^5^ ± 0.1 × 10^5^ T_H_ cells and 1.1 × 10^5^ ± 0.2 × 10^5^ T_R_ cells; 8.3 × 10^4^ ± 2.7 × 10^4^ T_H_::control cells and 5.1 × 10^4^ ± 0.9 × 10^4^ T_H_::Foxp3: cells; values ± SEM). Error-bars represent the SEM.

Encouraged by the initial finding that polyclonal CD4^+^CD25^−^ T cells transduced with Foxp3 (T_H_::Foxp3) can prevent and treat colitis in lymphopenic animals [19,29], we, like others [23,30,31], set out to test whether this can be used as a general strategy to prevent and treat autoimmune diseases. To test this hypothesis, we used collagen-induced arthritis (CIA), which is a well-established murine model of human rheumatoid arthritis [32]. To obtain T_H_::Foxp3 cells, we transduced CD4^+^CD25^−^ T cells with a murine leukemia virus (MLV)-based retroviral vector carrying a Foxp3-IRES-GFP cassette (m6pg[Foxp3]) (Figure S1). We immunized male DBA/1 mice with chicken collagen type II (cII) in complete Freund's adjuvant (CFA). In this model, we observe the first clinical symptoms of arthritis on day 19 after immunization, with the average clinical score reaching a plateau around day 35. Injection of 1 × 10^6^ T_H_::Foxp3 cells 1 d prior to immunization did not have any significant impact on the outcome of the arthritis. It neither delayed the time of disease onset, nor did it alter disease progression (Figure 1A). The failure of polyclonal T_H_::Foxp3 cells to show any beneficial effect on the outcome of CIA under these experimental conditions is in agreement with the findings of others [31] and led us to reassess the approach per se. Therefore, we decided to examine the homing, expansion, and participation of T_H_::Foxp3 cells in immune responses.

### Altered Homing Behavior of T_H_::Foxp3 Cells

The decision whether to launch or suppress an immune response is made within the secondary lymphoid organs [33]. This makes “correct” homing of the adoptively transferred cells an essential requirement for cytotherapy, as otherwise their participation in immune responses might be severely limited.

We therefore compared the homing of T_H_::Foxp3 cells to that of m6pg[control]-transduced CD4^+^CD25^−^ T (T_H_::control) cells (Figure S1) and freshly isolated CFSE-labeled CD4^+^CD25^−^ (T_H_) cells or CD4^+^CD25^+^ (T_R_) cells. A total of 1 × 10^6^ cells were injected into wild-type Balb/c mice. After 48 h, we isolated the lymphocytes from the various tissues and analyzed them by flow cytometry. The transferred cells were identified based on either their green fluorescent protein (GFP) coexpression or CFSE label. T_H_::control cells, like T_R_ and T_H_ cells, could be detected at comparable frequencies in blood, and inguinal and iliac lymph nodes, as well as the spleen (Figure 1B and 1C). In contrast, the homing of T_H_::Foxp3 cells into the lymph nodes appeared to be defective and their homing into the spleen slightly impaired. Instead, a large number of these cells could be found in the liver (Figure 1C). The data suggest that ectopic expression of Foxp3 substantially altered the homing behavior of the transduced cells.

### Foxp3-Mediated Regulation of CD62L

The absence of T cells from the peripheral lymph nodes is one of the key features of CD62L-deficient (*sell^−/−^*) mice [34]. CD62L (l-selectin) plays a key role in the homing of lymphocytes into these tissues by allowing their attachment to high endothelial venules [35]. Activation of T cells leads to endoproteolytic shedding of CD62L from the surface of the cells, involving the matrix-metalloprotease Adam17 [36]. Therefore, we investigated whether the altered homing behavior of T_H_::Foxp3 cells is due to Foxp3-mediated effects on the surface expression of CD62L.

**Figure 2:**
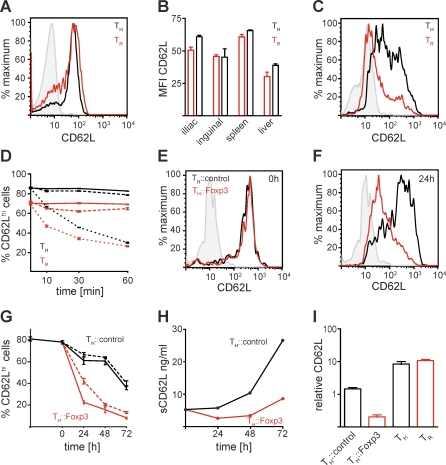
Foxp3-Mediated Regulation of CD62L (A–D) CD62L expression on CD4^+^Foxp3^−^ T_H_ cells (black) and CD4^+^Foxp3^+^ T_R_ cells (red). (A) Representative FACS profiles for CD62L expression on T_H_ and T_R_ cells prepared from spleen (*n* = 3 in each case) with unstained T_H_ cells (grey) shown as control. (B) Mean fluorescence intensity (MFI) of CD62L on T_H_ and T_R_ cells from indicated tissues (*n* = 2 in each case). (C) Representative FACS profiles of CD4^+^CD25^−^ T_H_ (black) and CD4^+^CD25^+^ T_R_ (red) cells activated for 72 h (*n* = 3 in each case). (D) Total splenocytes were incubated in the absence of any treatment (solid line) or activated by addition of 100 ng/ml PMA in the presence (dashed line) or absence (dotted line) of 50 μM TAPI-2 (*n* = 3 in each case). (E–I) CD62L expression in T_H_::control (black) and T_H_::Foxp3 cells (red). CD4^+^CD25^−^ cells were activated for 36 h and transduced (0 h) with either m6p8[control] (black line; *n* = 3) or m6p8[Foxp3] (red line; *n* = 3). (E and F) Representative FACS profiles of CD62L expression on transduced cells at (E) 0 h and (F) 24 h after transduction. (G) Percentage of CD62L^hi^ cells within the transduced populations in the presence (dashed line) or absence (solid line) of 50 μM TAPI-2. (H) Amount of soluble CD62L in the supernatant measured by ELISA (representative of two independent experiments). (I) Relative CD62L expression in CD4^+^CD25^−^ T_H_ and CD4^+^CD25^+^ T_R_ cells (*n* = 3 in each case), as well as T_H_::control and T_H_::Foxp3 cells 48 h after transduction (*n* = 2 in each case) determined by qPCR and normalized to HPRT. Error bars represent the SEM.

We found that the majority of freshly isolated T_H_ and T_R_ cells are CD62L^hi^ (Figure 2A and 2B). Activation of the cells for 72 h with anti-CD3/anti-CD28/IL-2 led to a down-regulation of CD62L surface expression, which was more marked in T_R_ than T_H_ cells (Figures 2C and S2A). To assess whether this is due to an increase in Adam17 activity in T_R_ cells, we activated freshly isolated splenocytes with PMA and compared the surface expression of CD62L on Foxp3^+^ (T_R_) and Foxp3^−^ (T_H_) CD4^+^ T cells. The rate of CD62L shedding appeared to be very similar for both cell types and could be completely blocked by the Adam17 inhibitor TAPI-2 (Figure 2D). This suggests that an additional Adam17-independent mechanism in T_R_ cells is responsible for the difference in CD62L surface expression observed upon activation of T_R_ and T_H_ cells.

To further investigate this, we examined CD62L expression in T_H_::Foxp3 cells. We transduced CD4^+^CD25^−^ cells with either m6p8[Foxp3] or m6p8[control]. The cells carrying the vector were identified based on their coexpression of ratCD8α (Figure S1). Whereas T_H_::control cells exhibited some down-regulation of surface CD62L upon activation with anti-CD3/IL-2, this was substantially more marked in T_H_::Foxp3 cells (Figure 2E and 2F). For the first 24 h, TAPI-2 appeared to partially inhibit the loss of surface CD62L on T_H_::Foxp3 cells, but it did not halt the steady decrease in surface CD62L over an extended period of time (Figure 2G). The CD62L down-regulation in T_H_::control cells was accompanied by an accumulation of soluble CD62L in the culture supernatant. This was not the case for T_H_::Foxp3 cells (Figure 2H), suggesting that in these cells, CD62L surface expression is regulated by a mechanism other than shedding. As Foxp3 is known to be a transcriptional regulator [37–40], we investigated whether it affects CD62L transcription. The CD62L mRNA expression level was reduced in both T_H_::Foxp3 and T_H_::control cells compared to freshly isolated T_H_ and T_R_ cells (Figure 2I). However, the level of CD62L transcript was 7.2-fold lower in T_H_::Foxp3 cells than in T_H_::control cells. The data suggest that upon activation of the cells, CD62L is further down-regulated on a transcriptional level by Foxp3.

It is noteworthy that retroviral transduction requires at least some degree of activation of the cell to allow for transgene integration. In this context, the expression of Foxp3 led to a very marked and sustained down-regulation of surface CD62L expression. This is likely to be a major contributor to the altered homing behavior of T_H_::Foxp3 cells. Although the down-regulation of CD62L upon activation is similarly more evident in thymically derived T_R_ cells than T_H_ cells (Figure S2A and S2B), albeit less marked than in T_H_::Foxp3 (Figure 2I), it does not appear to interfere with the cells ability to home into peripheral lymph nodes (Figure S2C).

### iFoxp3—an Engineered Inducible Lineage Factor

The “incorrect” homing of polyclonal T_H_::Foxp3 cells might well contribute to their lack of showing any beneficial effect in CIA [31] (Figure 1A) and other animal models of autoimmune disease [11]. However, one might question whether our initial approach had any merit in the first place, since the transfer of polyclonal T_H_::Foxp3 cells will only marginally increase the number of suppressive cells that recognize a particular antigen. Indeed, treatment with polyclonal T_H_::Foxp3 cells more or less mimics polyclonal T_R_ cell therapy, which in contrast to approaches using antigen-specific T_R_ cells, appears to be of limited benefit [22–24,41].

We decided to develop an alternative strategy, allowing us to convert the lineage commitment of conventional T_H_ cells to that of T_R_ cells after their antigen-specific expansion in vivo. To achieve this, we created an inducible Foxp3 (iFoxp3) that is constitutively expressed, but only becomes functionally active upon induction. Polyclonal, primary T_H_ cells transduced with iFoxp3 (T_H_::iFoxp3 cells) should act like conventional T cells, retain their homing behavior, participate in immune responses, and expand in an antigen-specific fashion. This antigen-specific in vivo expansion of T_H_::iFoxp3 cells should allow us to specifically switch off immune responses on demand by inducing iFoxp3.

**Figure 3:**
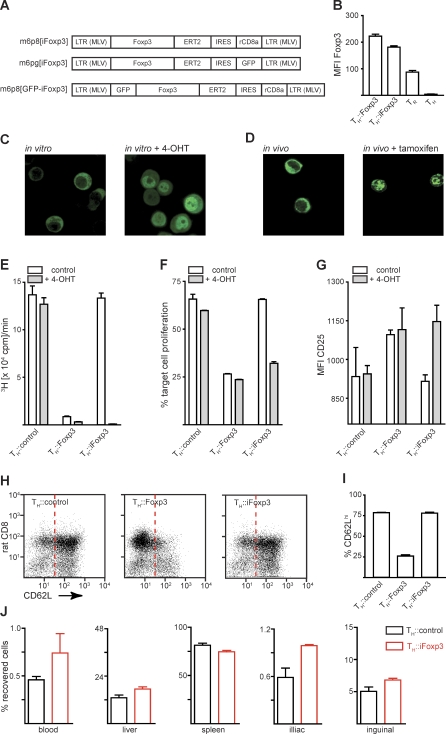
Inducible Foxp3 (A) Diagram of iFoxp3 containing retroviral vectors m6pg[iFoxp3] either coexpressing GFP or a GPI-linked ratCD8 α-chain m6p8[iFoxp3], and m6p8[GFP-iFoxp3], which contains a fusion of GFP and iFoxp3. (B) MFI of intracellular stain for Foxp3 in T_H_::Foxp3 and T_H_::iFoxp3 cells compared to CD4^+^ T_R_ and T_H_ cells (*n* = 2 in each case). (C and D) Subcellular localization of GFP-iFoxp3 in T_H_::GFP-iFoxp3 cells (C) in vitro after 48 h in the presence or absence of 50 nM 4-OHT or (D) in vivo after three injections of tamoxifen or carrier. (E–G) Gain of T_R_ cell function upon induction of iFoxp3. (E) Proliferation of T_H_::control, T_H_::Foxp3, and T_H_::iFoxp3 cells upon anti-CD3ɛ (0.6 μg/ml) stimulation measured by ^3^H-thymidine incorporation in the absence (white bars; *n* = 3 in each case) or presence of 50 nM 4-OHT (grey bars; *n* = 3 in each case). (F) A total of 1 × 10^5^ CFSE-labeled CD4^+^CD25^−^ target T cells were cocultured with 1 × 10^5^ T_H_::control, T_H_::Foxp3 or T_H_::iFoxp3 cells and activated with anti-CD3ɛ (0.6μg/ml) (*n* = 2 in each case). The proliferation of target cells was measured based on CFSE dilution after 72 h, and the percentage of cells that had undergone at least one cell cycle is shown. The assay was performed in the absence (white bars) or the presence (grey bars) of 50 nM 4-OHT added to the transduced cells 24 h prior to setup. (G) MFI of CD25 48 h after transduction on T_H_::control, T_H_::Foxp3, and T_H_::iFoxp3 in the absence (white bars; *n* = 2 in each case) or presence of 50 nM 4-OHT (grey bars; *n* = 2 in each case). (H and I) Comparison of CD62L expression on T_H_::control, T_H_::Foxp3, and T_H_::iFoxp3 48 h after transduction with m6p8. (H) Representative FACS profiles of CD62L expression (*n* = 2 in each case). (I) Percentage of CD62L^hi^ cells within the transduced populations. (J) Comparison of the homing behavior of T_H_::control (black) and T_H_::iFoxp3 (red) cells. A total of 1 × 10^6^ cells were transferred into each mouse (T_H_::control, *n* = 2; T_H_::iFoxp3, *n* = 3), and the tissues were analyzed 48 h later by flow cytometry. The diagrams represent the percentage of cells in each tissue calculated from the total number of cells recovered in all tissues together (5.4 × 10^5^ ± 0.7 × 10^5^ T_H_::control cells and 3.1 × 10^5^ ± 0.4 × 10^5^ T_H_::iFoxp3 cells; values ± SEM).

We fused a modified estrogen receptor (ERT2) to the C-terminal end of Foxp3 and cloned it into the m6p vector (Figure 3A and 3B). ERT2 only responds to tamoxifen and its metabolites such as 4-hydroxytamoxifen (4-OHT), but not estrogen [42]. In the absence of induction, iFoxp3 is retained in the cytoplasm and kept inactive by heat shock proteins binding to the ERT2 part of the fusion protein [43]. To confirm the inducible nature of iFoxp3, we transduced CD4^+^CD25^−^ cells with m6p carrying a GFP-tagged iFoxp3 (m6p8[GFP-iFoxp3]). This allowed us to assess the induction of iFoxp3 based on the translocation of the fusion protein from the cytoplasm into the nucleus. We induced iFoxp3 in vitro by exposure to 4-OHT for 48 h (Figure 3C) or in vivo after adoptive transfer of the transduced cells into wild-type Balb/c mice by intraperitoneal (i.p.) injections of tamoxifen (Figure 3D). In either case, iFoxp3 translocated into the nucleus in about 60%–70% of the transduced cells at the time of microscopic analysis, confirming its inducible nature.

### Induction of Suppressor Function in T_H_::iFoxp3 Cells

A key requirement for our strategy is that iFoxp3 can be used to induce T_R_ cell phenotype on demand. We therefore tested T_H_::iFoxp3 cells for hallmark features of T_R_ cells such as sustained up-regulation of CD25, in vitro anergy to anti-CD3-stimulation, and suppression of target cells [4] before and after induction of iFoxp3. Whereas T_H_::Foxp3 cells were anergic (Figure 3E), suppressed the proliferation of cocultured CD4^+^CD25^−^ cells (Figure 3F), and exhibited up-regulation of CD25 (Figure 3G), T_H_::iFoxp3 cells did so only after induction of iFoxp3 with 4-OHT. This demonstrates that, at least in vitro, T_H_::iFoxp3 cells appear to behave like conventional T_H_ cells and only assume the phenotype of T_R_ cells upon the induction of iFoxp3.

### T_H_::iFoxp3 Home Like Naive CD4^+^ T Cells

From our observations with T_H_::control cells, we already knew that transduction per se did not appear to alter the homing behavior of the cells (Figure 1B and 1C). Nevertheless, we wanted to verify that noninduced iFoxp3 neither changes the expression of CD62L nor significantly alters the homing behavior of the T_H_::iFoxp3 cells. We found that in the absence of iFoxp3 induction, CD62L expression remained unchanged in T_H_::iFoxp3 compared to T_H_::control cells (Figure 3H and 3I). This is in stark contrast to our observations made for T_H_::Foxp3 cells (Figure 2E to 2I). To assess the homing behavior of the cells, we used the same approach as described above. We found that the homing behavior of T_H_::iFoxp3 cells was comparable to that of T_H_::control cells (Figure 3J) and thus very similar to that of naive T_H_ and T_R_ cells (Figure 1B).

### Antigen-Specific In Vivo Expansion of T_H_::iFoxp3 Cells

**Figure 4:**
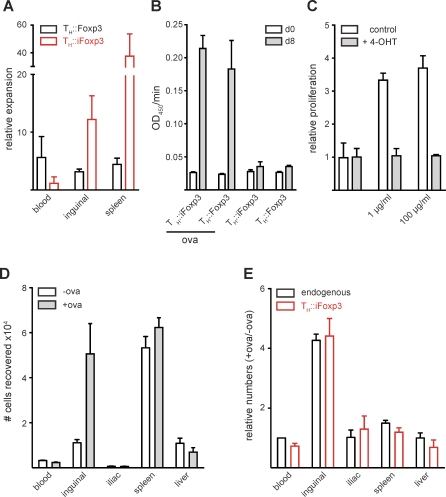
T_H_::iFoxp3 Cells Partake in the Immune Response and Suppress It upon Induction (A–C) Balb/c mice received 2 × 10^4^ T_H_::Foxp3 or T_H_::iFoxp3 cells prepared from DO11.10xSCID mice before being immunized s.c. with either ova in CFA (+ova) or CFA alone (−ova) (*n* = 3 in each case). (A) The frequency of GFP^+^ cells was measured eight days after immunization and the relative expansion was calculated as %GFP^+^ (+ova) / %GFP^+^ (-ova). (B) Total ova-specific antibodies in prebleeds (d0, white bars; *n* = 2 in each case) and 8 d after immunization (d8, grey bars; *n* = 3 in each case) in immunized and naive mice. (C) Total splenocytes were isolated from mice that had received T_H_::iFoxp3 cells and were challenged with the indicated amounts of ova for 72 h in the absence (white bars) or presence (grey bars) of 50 nM 4-OHT. The total proliferation was measured by ^3^H-thymidine incorporation, and the relative proliferation was calculated as (+ova / (−ova). (D and E) Mice received 1 × 10^6^ polyclonal T_H_::iFoxp3 cells and were immunized s.c. with ova in CFA. A week later, various tissues were analyzed. (D) The total number of recovered T_H_::iFoxp3 cells from immunized mice (red, *n* = 3) or nonimmunized mice (black, *n* = 3) was calculated. (E) The relative number of endogenous and T_H_::iFoxp3 cells was calculated as a ratio between immunized and nonimmunized mice. All error bars represent SEM, and *p*-values were determined using an unpaired *t*-test.

To assess whether T_H_::Foxp3 and T_H_::iFoxp3 cells expand upon antigenic challenge in vivo, we transferred transduced cells prepared from DO11.10xSCID/Balb/c mice that expressed an ovalbumin (ova)-specific TCR, into wild-type Balb/c mice. We transferred 5 × 10^4^ cells containing a mixture of 2 × 10^4^ T_H_::iFoxp3 cells and 3 × 10^4^ nontransduced cells (transduction efficiency of 40%) with the transduced population being clearly identifiable based on the coexpression of GFP. T_H_::iFoxp3 cells expanded upon immunization with ova in CFA by a factor of 12 in the draining lymph nodes and by a factor of 37.5 in the spleen (Figure 4A). In contrast, T_H_::Foxp3 cells only exhibited a very modest expansion, by a factor of 3.6 in the lymph nodes and 4.4 in the spleen. This could have been due to the T_H_::Foxp3 cells suppressing the ova-specific immune response and thereby impeding their own expansion. However, the levels of ova-specific antibodies in the serum were the same, independent of whether the mice had received T_H_::Foxp3 or T_H_::iFoxp3 cells, suggesting this was not the case (Figure 4B). Our data demonstrates a clear expansion of T_H_::iFoxp3 cells, which is consistent with their participation in the immune response against ova. This in vivo expansion upon antigen exposure is considerably less marked in T_H_::Foxp3 cells.

Next, we investigated whether the in vivo expanded ova-specific T_H_::iFoxp3 cells can be induced to suppress the very same immune response they partook in. We isolated splenocytes from these mice and exposed them to ova ex vivo. Although, in the absence of induction of iFoxp3, we observed the expected antigen-induced recall proliferation, we could not detect any proliferation above background in the presence of 4-OHT (Figure 4C). This suggests that upon iFoxp3 induction, the expanded T_H_::iFoxp3 cells became anergic and suppressed the proliferation of the cotransferred, nontransduced DO11.10 T cells as well as any endogenous ova-specific T cells.

To assess to what degree polyclonal T_H_::iFoxp3 cells participate in an immune response, we transferred 1 × 10^6^ wild-type T_H_::iFoxp3 cells into wild-type Balb/c mice. A week after immunization with ova, we analyzed the lymphocytes from various tissues by flow cytometry. Whereas the number of T_H_::iFoxp3 cells recovered from the blood, iliac lymph nodes, liver, and spleen did not appear to change upon antigenic challenge, we observed a marked increase in the inguinal lymph nodes of the immunized mice (Figure 4D). This indicates that some of the T_H_::iFoxp3 cells expanded in the draining lymph nodes (subcutaneous [s.c.] immunization into the flanks). However, the number of endogenous cells in the inguinal lymph nodes increased equally (Figure 4E), suggesting that both populations expand to a similar degree with their ratio remaining constant.

### Switching Off Immune Responses

**Figure 5:**
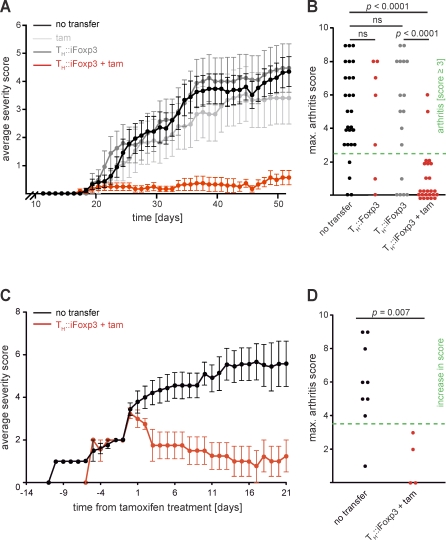
T_H_::iFoxp3 Cells Suppress Collagen-Induced Arthritis upon iFoxp3 Induction (A and B) Arthritis was induced on day 0 by immunization with cII in CFA. (A) Mice that received 1 × 10^6^ T_H_::iFoxp3 cells (dark grey, *n* = 17), mice that did not receive any further treatment (black, *n* = 27), mice that received tamoxifen injections (light grey, *n* = 14), and mice that received 1 × 10^6^ T_H_::iFoxp3 cells and tamoxifen injections to induce iFoxp3 (red, *n* = 25) are shown. The average arthritis scores of all mice in the groups are shown for each day. (B) Maximum arthritis score reached by individual animals that had received no transfer of cells, T_H_::Foxp3 cells (see Figure 1A), and T_H_::iFoxp3 cells ± tam. tam, tamoxifen. (C and D) Arthritis was induced by immunization with cII in CFA. (C) Mice that had received 1 × 10^6^ T_H_::iFoxp3 cells the day before cII immunization and tamoxifen injections (red, *n* = 4) when the mice reached a score of 3 (day 0) and mice that did not receive any further treatment (black, *n* = 9) are shown. (D) Maximum arthritis score reached by individual animals. Error bars represent the SEM, and *p*-values were determined by Fisher's Exact Test.

To test the potential of T_H_::iFoxp3 cells in suppressing autoimmune responses, we turned to the CIA model in which T_H_::Foxp3 cells had failed to show an effect (Figure 1A). We transferred 1–2 × 10^6^ polyclonal T_H_::iFoxp3 cells into wild-type DBA/1 mice 1 d prior to immunization with cII in CFA. We induced iFoxp3 on day 15 after immunization, which lies between the peak of the T cell response to collagen around day 10 [44,45] and the onset of clinical symptoms around day 21 [46]. Mice that had received T_H_::iFoxp3 cells but did not receive tamoxifen injections to induce iFoxp3 showed the first signs of arthritis on day 19, similar to the mice that received no transfer of cells (Figure 5A). This effect was specific to the antigenic challenge (cII in CFA) inducing the autoimmune response, as mice receiving these cells without immunization did not exhibit any overt signs of developing autoimmune disease (Figure S3). Remarkably, 23 out of 25 of the mice that had received T_H_::iFoxp3 cells and tamoxifen injections to induce iFoxp3 did not show any clinical signs of arthritis (scores <3; Figure 5B). This is in stark contrast to the other groups, in which the majority of animals developed arthritis (scores ≥3; Figure 5B). Whereas tamoxifen has been reported to have anti-inflammatory properties [47], we found that by itself, it had only a minor effect on the development of CIA (Figure 5A) and no effect on the activity of T_H_::control cells in vivo (Figure S4). Despite the clear suppression of the clinical signs of CIA, we could detect collagen-specific antibodies in the serum of the animals at day 52, irrespective of the treatment they had received (Figure S5).

Next, we investigated whether T_H_::iFoxp3 cells are capable of stopping already established CIA. To this end, we waited until the mice had reached a clinical score of 3 before inducing iFoxp3. The induction appeared to completely halt, if not reverse, CIA, leading to a decline in the average severity score (Figure 5C). None of the mice showed a further increase of symptoms after induction of iFoxp3 (Figure 5D).

### Specificity of the Suppression

**Figure 6:**
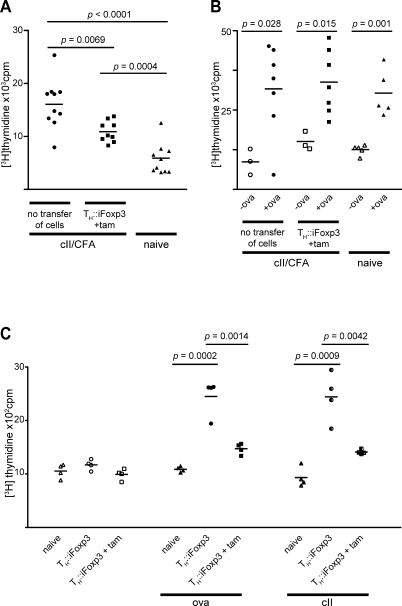
T_H_::iFoxp3 Cell-Mediated Suppression Is Specific (A and B) Mice were immunized with cII in CFA on day 0. tam, tamoxifen. (A) On day 35, ex vivo recall reactions to cII were performed on cells purified from mice that did not receive any further treatment (control, *n* = 10), mice that had received 1 × 10^6^ T_H_::iFoxp3 cells and tamoxifen injections (T_H_::iFoxp3 + tam, *n* = 10), and naive mice (naive, *n* = 10). (B) Some of the mice described in (A) were immunized on day 28 with ova, and ex vivo recall reactions to ova were performed in parallel (control, −ova: *n* = 3, +ova: *n* = 7; T_H_::iFoxp3 + tam, −ova: *n* = 3, +ova: *n* = 7; and naive, −ova: *n* = 5, +ova: *n* = 5). (C) Mice were immunized simultaneously with cII and ova in CFA on day 0. Ex vivo antigen-specific recall reactions to ova (closed) and cII (half-closed) were performed on day 28. Mice that did not receive any further treatment (naive, *n* = 4), mice that received 1 × 10^6^ T_H_::iFoxp3 cells and tamoxifen injections (T_H_::iFoxp3 + tam, *n* = 4), and mice that received 1 × 10^6^ T_H_::iFoxp3 cells (T_H_::iFoxp3, *n* = 4) are shown. *p*-Values were determined using an unpaired *t*-test.

To assess whether the conversion of T_H_::iFoxp3 cells to T_R_ cell phenotype causes systemic immunosuppression, we compared “ex vivo recall reactions” to the antigen used prior to the induction of iFoxp3 (cII) to that of an unrelated antigen (ova) injected after induction. The collagen-specific T cell proliferation measured for mice in which iFoxp3 had been induced was significantly lower than that of mice that had received no transfer of cells, albeit still higher than that of naive mice (Figure 6A). As we did not add tamoxifen to the ex vivo culture, this most likely reflects a lower number of cII-specific proinflammatory T cells in the animals that had received T_H_::iFoxp3 cells and tamoxifen induction, rather than a mere ex vivo suppressive effect of T_H_::iFoxp3 cells. Remarkably, we could not detect any difference in the T cell proliferation upon exposure to ova irrespective of whether the mice had received treatment or not (Figure 6B). This suggests that the suppression only affects immune responses in which the T_H_::iFoxp3 cells have had the opportunity to participate prior to induction of iFoxp3. Indeed, we were able to detect T_H_::iFoxp3 cells in the inflamed paw of cII-immunized mice, suggesting that in the absence of induction these cells can contribute to the inflammation (Figure S6) However, once converted, the T_H_::iFoxp3 cells, despite still being present (Figure S7A and S7B), seem to have lost the capacity to suppress further unrelated immunological challenges (Figure 6B). This suggests that the conversion of T_H_::iFoxp3 cells by induction of iFoxp3 does not lead to a systemic immunosuppression.

Having shown that induced T_H_::iFoxp3 cells do not suppress further unrelated immune responses postinduction, we wanted to investigate the suppressive activity of T_H_::iFoxp3 cells in a context in which both cII and ova are present prior to induction. We transferred 1 × 10^6^ polyclonal T_H_::iFoxp3 cells into wild-type DBA/1 mice 1 d before immunization with a 1:1 mixture of ova and cII in CFA. We induced iFoxp3 on day 15 after immunization and assessed the antigen-induced proliferation of splenocytes prepared from these mice on day 28. The recall proliferation against ova and cII were comparable. Equally similar was the reduction in proliferation in the cases in which iFoxp3 was induced (Figure 6C). In combination, these results suggest that this approach enables selective suppression without affecting further unrelated immune responses after induction of iFoxp3.

### T_H_::iFoxp3 Cell Longevity

**Figure 7:**
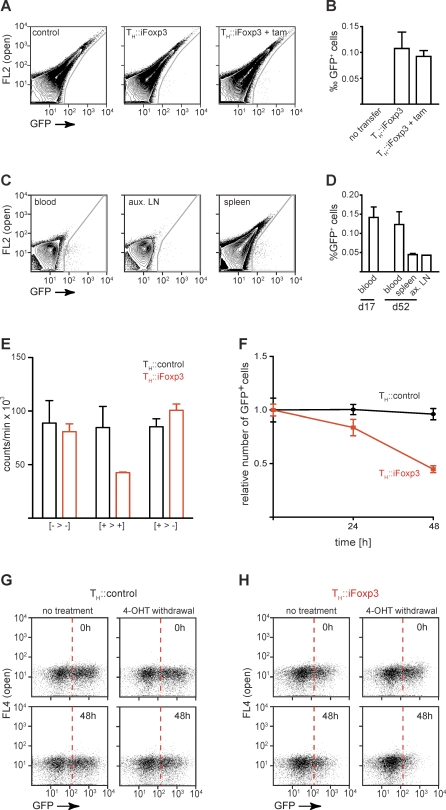
T_H_::iFoxp3 Cell Longevity (A) Representative FACS profiles of splenocytes purified from the indicated mice 52 d after transfer of 1 × 10^6^ T_H_::iFoxp3 cells. tam, tamoxifen. (B) Summary of the frequency of GFP^+^ cells in the spleen 52 d after transfer (*n* = 3 in each case). (C) Representative FACS profiles of specified tissues 52 d after transfer of 2 × 10^6^ T_H_::iFoxp3 cells (*n* = 4 in each case; for auxiliary lymph node [aux. LN], a pooled sample was analyzed). (D) Summary of the frequency of T_H_::iFoxp3 cells in the various tissues 17 and 52 d after transfer. (E–H) T_H_::iFoxp3 cell survival upon 4-OHT withdrawal (E). T_H_::control and T_H_::iFoxp3 were cultured in the continuous presence [+ > +] or absence [− >−] of 50 nM 4-OHT. In the case of [+ >−], 4-OHT was withdrawn for 72 h after an initial induction for 48 h, before their suppressive activity was measured. A total of 1 × 10^5^ cells of the indicated populations were cocultured at a 1:1 ratio with 1 × 10^5^ CD4^+^CD25^−^ target cells in 96-well plates coated with anti-CD3ɛ [0.6 μg/ml]. The proliferation of the cells was measured after 72 h based on ^3^H-thymidine incorporation (*n* = 3 in each case). (F–H) T_H_::control and T_H_::iFoxp3 were cultured in the presence or absence of 4-OHT (50 nM) and anti-CD3ɛ (0.6 μg/ml). After 48 h, 4-OHT and anti-CD3ɛ was withdrawn. The viability of the cells was assessed by flow cytometry at 0 h, 24 h, and 48 h by measuring the coexpression of GFP. (F) Ratio of cells after 4-OHT withdrawal and cells that were cultured in the absence of 4-OHT from the start. (G and H) Representative FACS profiles of T_H_::control and T_H_::iFoxp3 cells. All error bars represent the SEM.

It is noteworthy that we were able to detect T_H_::iFoxp3 cells 52 d after their transfer, independent of the level of arthritis and whether the mice had received tamoxifen treatment or not (Figure 7A and 7B). An analysis of various tissues revealed that T_H_::iFoxp3 cells in blood were only marginally reduced between day 17 and day 52 (Figure 7C and 7D), and could readily be detected in the auxiliary lymph nodes and spleen. Although this is likely to be of advantage with regard to actively suppressing immune responses, it poses the question whether continuous tamoxifen presence is required. Due to the long half-life of tamoxifen [48], a direct assessment of this in vivo is not feasible. However, in in vitro suppression assays, T_H_::iFoxp3 cells had completely lost their suppressive activity 72 h after withdrawal of 4-OHT (Figure 7E). To perform these experiments, we had to compensate for a marked reduction in the number of viable T_H_::iFoxp3 cells that could be recovered under these conditions. To formally address the effect of the withdrawal of 4-OHT on T_H_::iFoxp3 cell viability, we exposed the cells to 4-OHT for 48 h from the point of transduction and then cultured them for a further 48 h in the absence of 4-OHT. The number of viable cells was assessed by flow cytometry. Withdrawal of 4-OHT had no effect on T_H_::control cells, but led to a marked decrease in the number of T_H_::iFoxp3 cells (Figure 7F to 7H). This suggests, that once induced, T_H_::iFoxp3 cells die upon tamoxifen withdrawal, but it remains unclear how this translates into an in vivo context. Indeed, it might be desirable to incorporate a suicide gene [49] into the retroviral vector as this allows the removal of the transduced cells if desired (Figure S8).

## Discussion

Here, we have presented an approach that allows us to stop undesirable immune responses without prior knowledge of the antigens involved. T_H_::iFoxp3 cells participate in immune responses as conventional T_H_ cells until iFoxp3 is induced. At this point, they change their phenotype from that of proinflammatory T cells to that of regulatory T cells and suppress the response they partook in.

Ectopic expression of Foxp3 in conventional T cells leads to their conversion into cells with T_R_-like phenotype [19–21]. It was demonstrated early on that these T_H_::Foxp3 cells, like T_R_ cells, could suppress the development of colitis in lymphopenic hosts [19,29]. However, it was noted that in this context, the effectiveness of both polyclonal T_H_::Foxp3 cells and T_R_ cells [29,50,51] might be due to the regulation of homeostatic expansion of the cotransferred, proinflammatory cells, rather than to a true antigen-specific suppression [9,11,52]. Furthermore, adoptive transfer of polyclonal T_R_ cells will only marginally increase the number of suppressive cells that recognize a particular antigen. Indeed, the use of polyclonal T_R_ cell [22] or T_H_::Foxp3 populations [11,23] (Figure 1A) have been of limited efficacy, unless the immune pathology was caused by an absence of functional T_R_ cells [20,53] or the experiments were performed in lymphopenic animals [11]. The restrictions imposed by the low frequency of antigen-specific T_R_ or T_H_::Foxp3 cells in polyclonal populations can be circumvented by ex vivo expansion of antigen-specific T_R_ cells and TCR transgenic T_H_::Foxp3 cells [9–11,41]. Both approaches have been successfully exploited in mouse models to treat diabetes [23,24,54,55], arthritis [31], and experimental autoimmune encephalomyelitis (EAE) [56], as well as being used for the induction of transplantation tolerance [57,58]. Whereas TCR transgenic T cells are an invaluable research tool to improve our understanding of the regulation of immune responses [59,60], it is unclear to what degree they can be used in a therapeutic context. Ex vivo expansion of antigen-specific T_R_ cells [9,11], or in vivo conversion of T_H_ into T_R_ cells [12], promises to be more applicable. However, these approaches are technically challenging, time consuming, and most importantly, require knowledge of or access to the antigens involved in the immune response to be suppressed [8,13].

Our study of T_H_::Foxp3 cells revealed a further problem. Whereas T_H_::Foxp3 cells appear to adopt the characteristics of T_R_ cells in vitro, we found their homing to be altered from that of endogenous T_R_ and T_H_ cells. This hinders the T_H_::Foxp3 cells from mimicking the homing behavior of endogenous T_R_ cells, which has been shown to be important for their suppressive function in vivo [61–63]. Those T_H_::Foxp3 cells that fail to home to the secondary lymphoid organs might not receive the required antigen priming [63] and thus fail to expand like endogenous T_R_ cells [64]. This might explain the difference in the efficacy of approaches that use polyclonal Foxp3^+^ cells and those that use antigen-selected or TCR transgenic Foxp3^+^ cells. The latter might circumvent the need for an antigen-specific expansion in vivo by ensuring that there are sufficient numbers of antigen-specific cells from the onset.

The activation-induced, Foxp3-mediated down-regulation of CD62L might well be a key factor in the exclusion of T_H_::Foxp3 cells from the peripheral lymph nodes since T cells from CD62L-deficient mice exhibit a similar phenotype [34,35]. Further, it has been shown that CD62L^hi^ polyclonal T_R_ cells have a more potent protective effect in vivo [65]. However, we cannot exclude that ectopic expression of Foxp3 also alters the expression of other homing receptors. Indeed, we found that the activation-induced down-regulation of CD62L in thymically derived T_R_ and T_H_ cells was not sufficient to exclude them from the peripheral lymph nodes.

Here, we present an approach that addresses these problems by transducing polyclonal, conventional T cells with a retroviral vector encoding a genetically engineered inducible form of Foxp3. T_H_::iFoxp3 cells retain their proinflammatory character and the ability to home to the lymph nodes. Those T_H_::iFoxp3 cells that recognize an antigen appear to participate in the immune response and expand. This in vivo expansion of antigen-specific T_H_::iFoxp3 cells circumvents the need for an ex vivo expansion and does not rely on any knowledge of the antigens involved. Upon induction of iFoxp3, the in vivo expanded, antigen-specific T_H_::iFoxp3 cells assume a T_R_ cell-like phenotype and suppress the undesirable response they initially partook in. We were able to demonstrate the efficacy of our approach by specifically halting CIA in a mouse model. Importantly, T_H_::iFoxp3 cell-mediated suppression appears to be restricted to the specific response, which is ongoing at the time of induction of iFoxp3. Those T_H_::iFoxp3 cells that do not already participate in an immune response at the time of induction lose the capacity to suppress further unrelated immune responses despite still being present. Although we cannot exclude that other factors play a role, it appears that the antigen-specific expansion of the T_H_::iFoxp3 cells prior to induction is an integral part of the observed nonsystemic suppression. In a therapeutic context, it might be desirable to limit the exposure to tamoxifen to minimize possible side effects. Although it appears that most T_H_::iFoxp3 cells die upon withdrawal of tamoxifen, those that do survive lose their suppressive activity. To avoid possible deleterious effects, these “revertant” cells can be removed based on the incorporation of a suicide gene into the retroviral vector used for the delivery of iFoxp3.

We believe that this strategy of induced conversion of T_H_ cells into cells with a T_R_ cell-like phenotype using iFoxp3 is generally applicable and will allow us to stop a variety of undesirable immune responses.

## Materials and Methods

### Animals and cell preparations.

Balb/c and DBA/1 mice (8–12 wk old) were purchased from Charles River and Harlan. DO11.10xSCID mice on the Balb/c background were kindly provided by Caetano Reis e Sousa, CRUK. Animals were maintained under specific pathogen-free conditions. Expert animal technicians provided animal care in compliance with the relevant laws and institutional guidelines. Cells used for in vivo and ex vivo experiments were purified (>90% purity) using an AutoMACS (Miltenyi Biotec) as previously described [66]. Flow cytometric analysis and proliferation assays were performed as described previously [66] using the following antibodies: ratCD8α (BD Bioscience), CD62L (BD Bioscience), CD4 (BD Bioscience), CD25 (BD Bioscience), and Foxp3 (eBioscience).

### Retroviral vectors and transduction.

Foxp3 was amplified from total spleen cDNA and iFoxp3 was constructed by a C-terminal fusion of ERT2 in place of the stop codon. Both were cloned into m6p retroviral vectors coexpressing either GFP or a GPI-linked rat CD8α marker. For the measurement of in vivo translocation of iFoxp3, GFP was cloned in-frame with Foxp3 after the first five codons in the 5′-end [67] in order to produce GFP-iFoxp3. For the production of retroviral supernatant, 293eT cells were cotransfected with an equal amount of pCl-Eco packaging plasmid and the respective m6p retroviral construct. Supernatant was harvested at 36 h and 48 h after transfection, filtered, and then used immediately. For retroviral transduction, the freshly purified CD4^+^CD25^−^ T cells were activated in the presence of plate-bound anti-CD3ɛ (0.6 μg/ml) (BD Bioscience) and 10 U/ml of recombinant mIL-2 (PeproTech). Cells were transduced at 24 h and 36 h after activation by resuspension in a 1:2 mixture of supernatant and complete medium (RPMI/10% FCS/10 μM β-mercaptoethanol/50 μg/ml gentamicin) supplemented with 10 U mIL-2 and 6 μg/ml protamine sulphate (Sigma) and 10 U/ml mIL2, followed by centrifugation at 600×*g* for 2 h at 32 °C. Six hours after transduction, cells were resuspended in complete medium containing 10 U mIL-2. A fixed ratio of transduced (50%–60% in all cases) and nontransduced cells was adoptively transferred into mice 72 h after the last transduction.

### Collagen-induced arthritis and gene induction.

Male DBA/1 mice received 1–2 × 10^6^ transduced cells intravenously (i.v.) (day −1) and were immunized intradermally (i.d.) with 100 μl of cII (Sigma,) dissolved in 10 mM acetic acid and emulsified (1 μg/μl) in CFA (DIFCO) the following day (day 0) [46]. The mice were assessed (blinded) on a daily basis, and inflammation of the paws was scored as follows: grade 0—no swelling; grade 1—swelling in an individual joint; grade 2—swelling in more than one joint or mild inflammation of the paw; and grade 3—severe swelling of the entire paw and/or ankylosis. Each paw was graded, and all scores where totaled for a maximum score of 12 per mouse. Mice reaching a score of 8 or more were euthanized in accordance with restrictions imposed by UK legislation. For iFoxp3 induction, the mice were injected i.p. with 100 μl of tamoxifen (in a 10:1 mixture of sunflower oil:ethanol [10 μg/μl tamoxifen]) on days 15 and 16, and (in a 10:1 mixture of sunflower oil:ethanol [1 μg/μl tamoxifen]) on days 23, 29, 30, 36, and 43. Alternatively, iFoxp3 was induced once the mice had reached a score of 3 (day 0) by i.p. injections with 100 μl of tamoxifen (in a 10:1 mixture of sunflower oil:ethanol [10 μg/μl tamoxifen]) on days 1, 2, 9, and 16.

### In vivo expansion of antigen-specific T cells and ova-specific suppression assay.

CD4^+^CD25^−^ T cells were purified from 6–12-wk-old female SCIDxDO11.10 mice and transduced with Foxp3 or iFoxp3 as described above. Balb/c females received i.v. 5 × 10^4^ of a 2:3 ratio of transduced and nontransduced cells. Three days later, each mouse was immunized s.c. with either ova (Sigma) in CFA (50 μg/mouse) or just with CFA. The mice were sacrificed and analyzed 8 d after immunization. For ova-specific suppression assays, total splenocytes were prepared as described [66], resuspended in complete medium, and then plated into round-bottom 96-well plates (density of 2 × 10^5^ cells/well). iFoxp3 was induced by adding 50 nM 4-OHT (Sigma). Ova was added to the cells 16 h after induction. After 60 h, the cells were pulsed with 1 μCi of ^3^H-thymidine (Amersham), collected at 72 h with a Filtermate Harvester (Packard), and analyzed with a TopCount scintillation counter (Packard) according to the manufacturer's instructions.

### Collagen- and ova-specific ex vivo recall reactions.

CIA and iFoxp3 induction was performed as described above. On day 28, some of the mice received ova in CFA s.c. into both flanks (100 μg/mouse). Total splenocytes were prepared on day 35 and plated into round-bottom 96-well plates at a density of 5 × 10^5^ cells/well. Proliferation of the cells was measured 72 h after addition of either ova (100 μg/ml) or cII (100 μg/ml) as described above. Alternatively, mice were immunized simultaneously with ova and cII on day 0 by i.d. injection of a mixture of 100 μg of ova and 100 μg of cII in CFA. Recall reactions were performed on day 28 as described above at a density of 2 × 10^5^ cells/well.

### Elisa for the detection of collagen- and ova-specific antibodies.

Ninety-six–well flat-bottom plates (Nunc) were coated with either ova (50 μg/ml) or cII (2 μg/ml) at 4 °C for 16 h and blocked with 1% BSA in PBS for 1 h. A total of 50 μl of serial dilutions (starting at 1:50 for ova and 1:10,000 for cII) of mouse sera in PBS were incubated for 2 h. Biotin-conjugated IgG1, IgG2a, IgG2b, and IgG3 (BD Bioscience) were then applied for 2 h. For ova detection, IgM (BD Bioscience) was also included. The development of cII and ova-specific immunoglobulins was then measured using a DuoSet kit (R&D Systems) according to the manufacturer's instructions.

### Real-time reverse transcriptase PCR.

Total RNA was extracted using an RNeasy kit (Qiagen) including DNaseI treatment (Invitrogen). cDNA was synthesized with Superscript II (Invitrogen) with random hexamer primers (Amersham) following the manufacturers instructions. Real-time PCR was performed using Taqman SYBR green PCR master mix (Applied Biosystems) with primers specific for *Sell* (CD62L) and *Hprt*. The sequences used were: *Sell* primers: 5′-ATG CAG TCC ATG GTA CCC AAC TCA-3′ and 5′-CTG CAG AAA CAC AGT GTG GAG CAT-3′; *Hprt* primers: 5′-TTA AGC AGT ACA GCC CCA AAA TG-3′ and 5′-CAA ACT TGT CTG GAA TTT CAA ATC C-3′. An ABI Prism 7900 sequence detection system (Applied Biosystems) was used for 45 cycles of PCR according to the manufacturer's instructions.

## Supporting Information

Figure S1 Foxp3 and Control Retroviral Vectors Diagram of Foxp3 containing retroviral vectors either coexpressing GFP (m6pg[iFoxp3]) or a GPI-linked ratCD8 α-chain (m6p8[iFoxp3]), and retroviral vectors containing blasticidine-*S*-deaminase (bsd) as a control gene either coexpressing GFP (m6pg[control]) or a GPI-linked ratCD8 α-chain (m6p8[control]).
(114 KB PDF)

Figure S2 Activation-Mediated Down-Regulation of CD62L in T Cells (A and B) CD62L expression on CD4^+^CD25^−^ T_H_ cells (black) and CD4^+^CD25^+^ T_R_ cells (red). (A) Representative fluorescence-activated cell sorter (FACS) profiles for CD62L expression on T_H_ and T_R_ cells prepared from spleen (*n* = 2) and activated with αCD3ɛ, αCD28, and IL-2 for the indicated length of time. (B) Representative graph of the relative mRNA levels of *Sell* (CD62L) in CD4^+^CD25^−^ T_H_ and CD4^+^CD25^+^ T_R_ cells activated for the indicated length of time (*n* = 2) determined by quantitative PCR (qPCR) and normalized to *Hprt*.
(C) Comparison of the homing behavior of activated m6pg[control]-transduced CD4^+^CD25^−^ T_H_ (black, *n* = 8) and CD4^+^CD25^+^ T_R_ (red, *n* = 8) cells. A total of 1 × 10^6^ cells were transferred into each mouse, and the tissues were analyzed 48 h later by flow cytometry as described above.
(408 KB PDF)

Figure S3 Adoptive Transfer of T_H_::iFoxp3 Cells Does Not Lead to any Overt Signs of Autoimmune Disease Balb/c mice received 2 × 10^6^ T_H_::iFoxp3 cells (red, *n* = 7) or no cells (black, *n* = 5) and were visually inspected and weighed weekly for 11 wk.
(259 KB PDF)

Figure S4 Tamoxifen Treatment Has No Effect on T_H_::Control Cells In Vivo Total splenocytes were isolated from mice that had received no transfer of cells or 1 × 10^6^ polyclonal T_H_::control and were challenged with ova in CFA. Some of the mice were injected with tamoxifen on day 4 after immunization (*n* = 3 in all cases). The relative proliferation is shown as a ratio of thymidine incorporation in the presence or absence of ova stimulation in the recall reaction performed on day 7. All error bars represent the standard error of the mean (SEM), and the *p*-values were determined using an unpaired *t*-test.
(276 KB PDF)

Figure S5 Level of Collagen-Specific IgG Antibodies Levels of collagen-specific IgG1, IgG2a, IgG2b, and IgG3 on day −2 and 52 in control mice (black, *n* = 6) and mice that had received T_H_::iFoxp3 cells and tamoxifen injections (red, *n* = 6). All error bars represent the SEM.
(131 KB PDF)

Figure S6 Migration of T_H_::iFoxp3 Cells into the Inflamed Paw Mice received either 1 × 10^6^ T_H_::iFoxp3 cells or no cell transfer (*n* = 2 in both cases). Arthritis was induced on day 0 by immunization with cII in CFA. The front and hind paws of arthritic mice were dissected on day 45, and the GFP^+^ cells were detected by flow cytometry. Error bars represent the SEM.
(260 KB PDF)

Figure S7 Survival of T_H_::iFoxp3 Cells in the Presence or Absence of Antigen Mice received 1 × 10^6^ polyclonal T_H_::iFoxp3 cells on day 0 and were immunized with ova as indicated on day 5. Some of the mice also received tamoxifen injections either on day 0 or day 8. The number of T_H_::iFoxp3 cells present in the spleen was assessed by flow cytometry based on GFP expression on day 13.
(A) Representative FACS profiles.
(B) Summary of the relative number of GFP^+^ cells in the spleen normalized to the total number of recovered cells (*n* = 3 in absence and *n* = 4 in the presence of ova immunization). All error bars represent the SEM.
(218 KB PDF)

Figure S8 In Vivo Depletion of T_H_::GFP/TK Cells CD4^+^CD25^−^ T cells were transduced with a retroviral vector containing GFP coexpressing a herpes simplex thymidine kinase gene (m6ptk[GFP]). Twenty-four hours after transduction, 1 × 10^6^ cells were transferred into wild-type mice (day 0). Ganciclovir (1 mg/mouse) was administered for three consecutive days by i.p. injection; and on day 5, the inguinal lymph nodes and spleen were analyzed for the presence of T_H_::GFP/TK cells (*n* = 4 in all cases). All error bars represent the SEM.
(117 KB PDF)

## Abbreviations

4-OHT: 4-hydroxytamoxifen
CFA: complete Freund's adjuvant
cII: chicken collagen type II
CIA: collagen-induced arthritis
ERT2: mutated estrogen receptor sensitive to tamoxifen, but not estrogen
FACS: fluorescence-activated cell sorter
Foxp3: Forkhead box p3
GFP: green fluorescent protein
i.p.: intraperitoneal
iFoxp3: inducible Foxp3
ova: ovalbumin
s.c.: subcutaneous
SEM: standard error of the mean
T_H_: helper T cell
T_H_::iFoxp3: CD4^+^CD25^−^ T cell transduced with iFoxp3
T_H_::Foxp3: CD4^+^CD25^−^ T cell transduced with Foxp3
T_H_::control: CD4^+^CD25^−^ T cell transduced with a control gene
T_R_: regulatory T cell
