# Supplementary material for: Specific Immunosuppression with Inducible Foxp3-Transduced Polyclonal T cells
Source: PLoS Biol. 2008 Nov 11;6(11):e276. doi: 10.1371/journal.pbio.0060276 (PMC2581628; doi:10.1371/journal.pbio.0060276)
Supplement: Supplementary file 1 [file pbio.0060276.sg001.pdf]

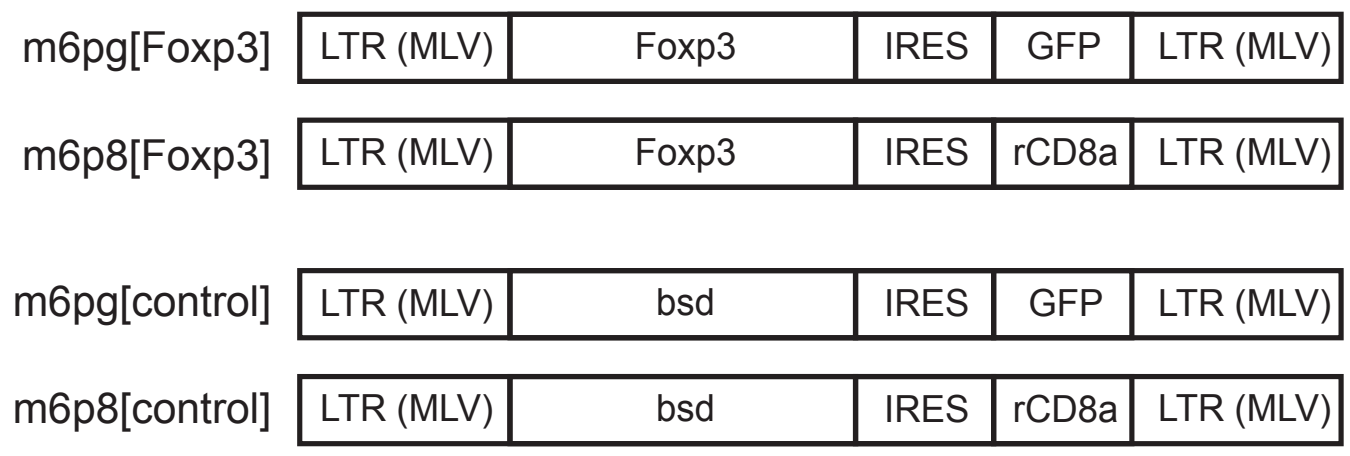
m6pg[Foxp3]
LTR (MLV) | Foxp3 | IRES | GFP | LTR (MLV)
m6p8[Foxp3]
LTR (MLV) | Foxp3 | IRES | rCD8a | LTR (MLV)
m6pg[control]
LTR (MLV) | bsd | IRES | GFP | LTR (MLV)
m6p8[control]
LTR (MLV) | bsd | IRES | rCD8a | LTR (MLV)
