# Supplementary material for: Specific Immunosuppression with Inducible Foxp3-Transduced Polyclonal T cells
Source: PLoS Biol. 2008 Nov 11;6(11):e276. doi: 10.1371/journal.pbio.0060276 (PMC2581628; doi:10.1371/journal.pbio.0060276)
Supplement: Supplementary file 2 [file pbio.0060276.sg002.pdf]

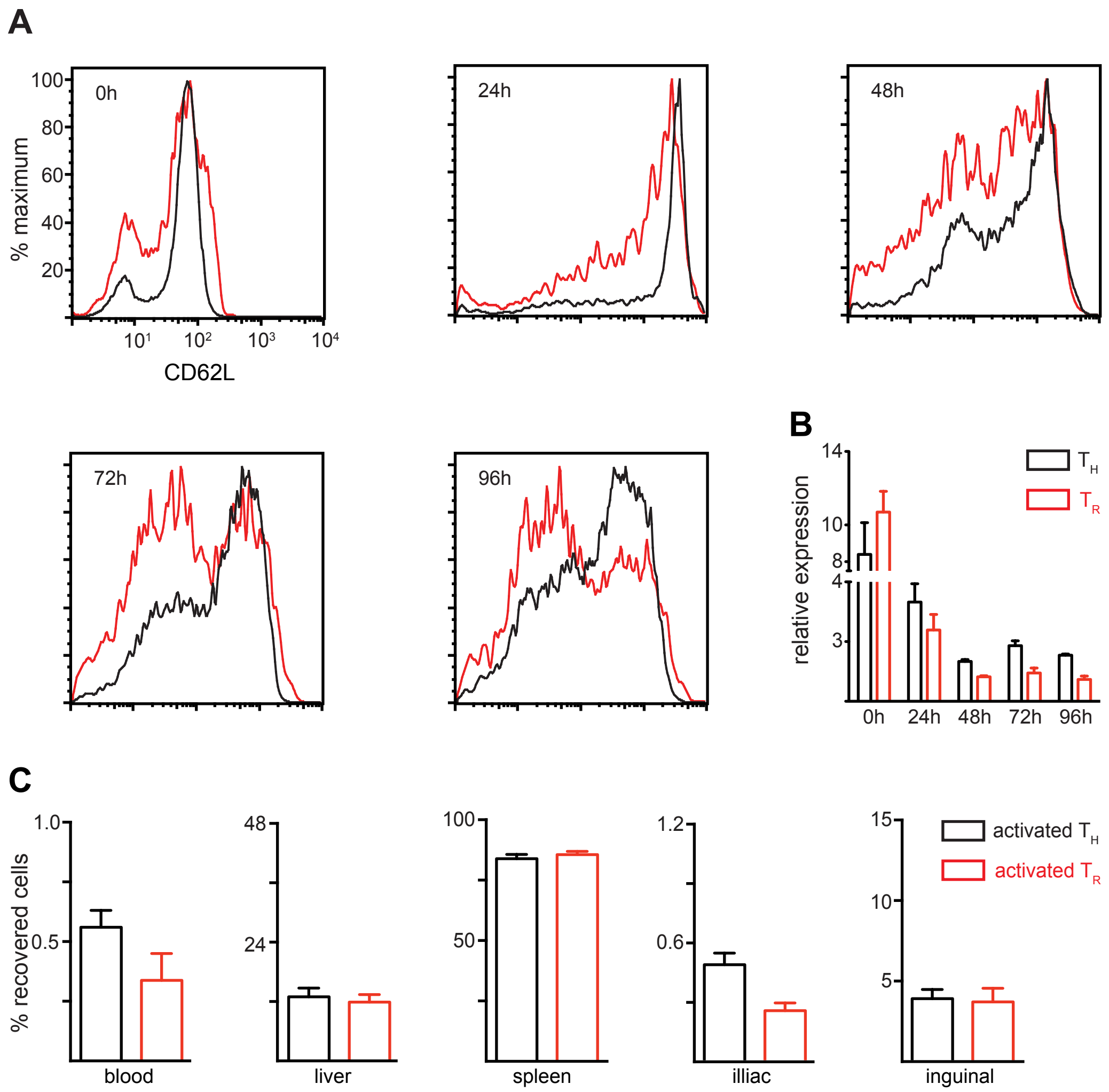

A
0h
24h
48h
72h
96h
% maximum
100
80
60
40
20
10^1
10^2
10^3
10^4
CD62L
B
relative expression
14
10
8
4
3
0h
24h
48h
72h
96h
T_H
T_R
C
% recovered cells
1.0
0.5
blood
48
24
liver
100
50
spleen
1.2
0.6
illiac
15
10
5
inguinal
activated T_H
activated T_R
