# Supplementary material for: Specific Immunosuppression with Inducible Foxp3-Transduced Polyclonal T cells
Source: PLoS Biol. 2008 Nov 11;6(11):e276. doi: 10.1371/journal.pbio.0060276 (PMC2581628; doi:10.1371/journal.pbio.0060276)
Supplement: Supplementary file 3 [file pbio.0060276.sg003.pdf]

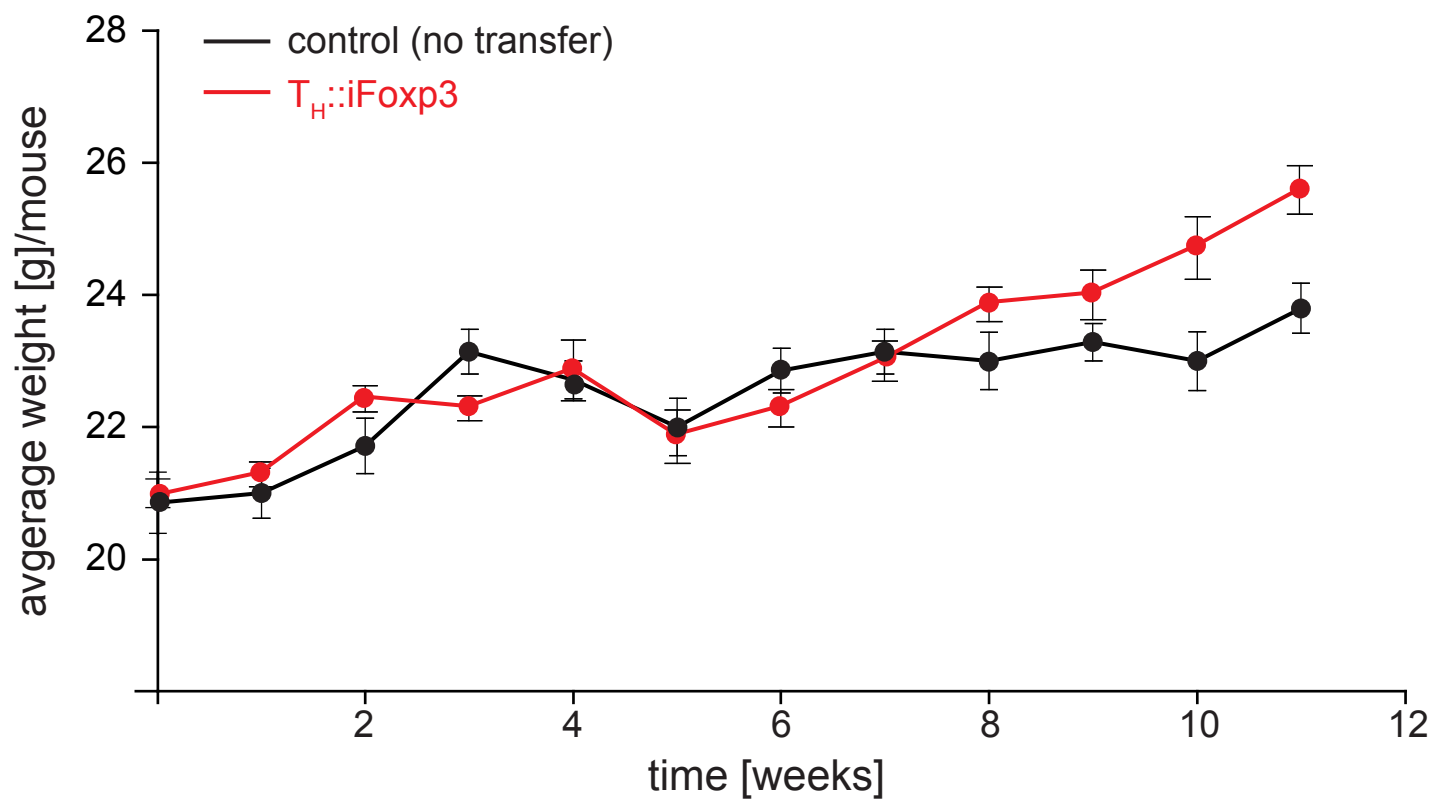

control (no transfer)
T_H::iFoxp3
avgerage weight [g]/mouse
28
26
24
22
20
2
4
6
8
10
12
time [weeks]
