# Supplementary material for: Specific Immunosuppression with Inducible Foxp3-Transduced Polyclonal T cells
Source: PLoS Biol. 2008 Nov 11;6(11):e276. doi: 10.1371/journal.pbio.0060276 (PMC2581628; doi:10.1371/journal.pbio.0060276)
Supplement: Supplementary file 4 [file pbio.0060276.sg004.pdf]

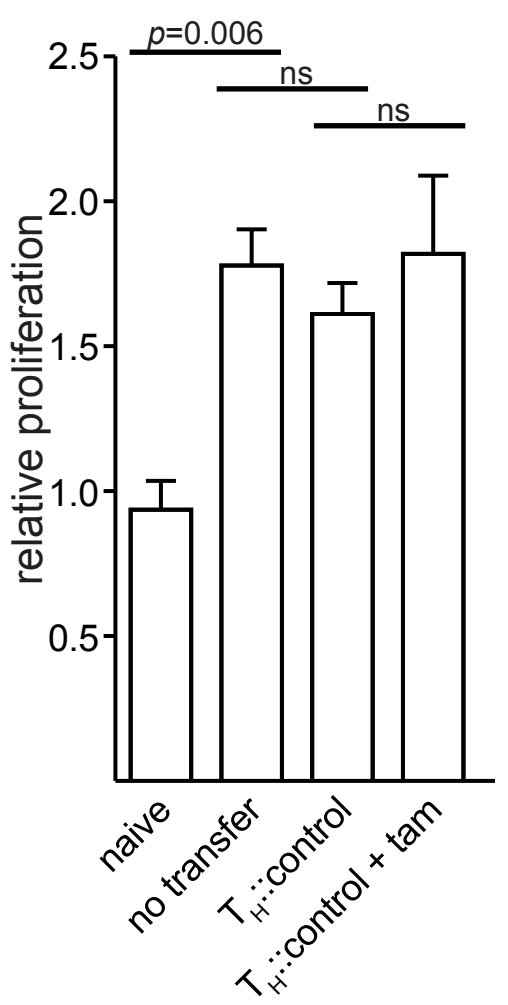
p=0.006
ns
ns
2.5
2.0
1.5
1.0
0.5
relative proliferation
naive
no transfer
T_H::control
T_H::control + tam
