# Supplementary material for: Specific Immunosuppression with Inducible Foxp3-Transduced Polyclonal T cells
Source: PLoS Biol. 2008 Nov 11;6(11):e276. doi: 10.1371/journal.pbio.0060276 (PMC2581628; doi:10.1371/journal.pbio.0060276)
Supplement: Supplementary file 5 [file pbio.0060276.sg005.pdf]

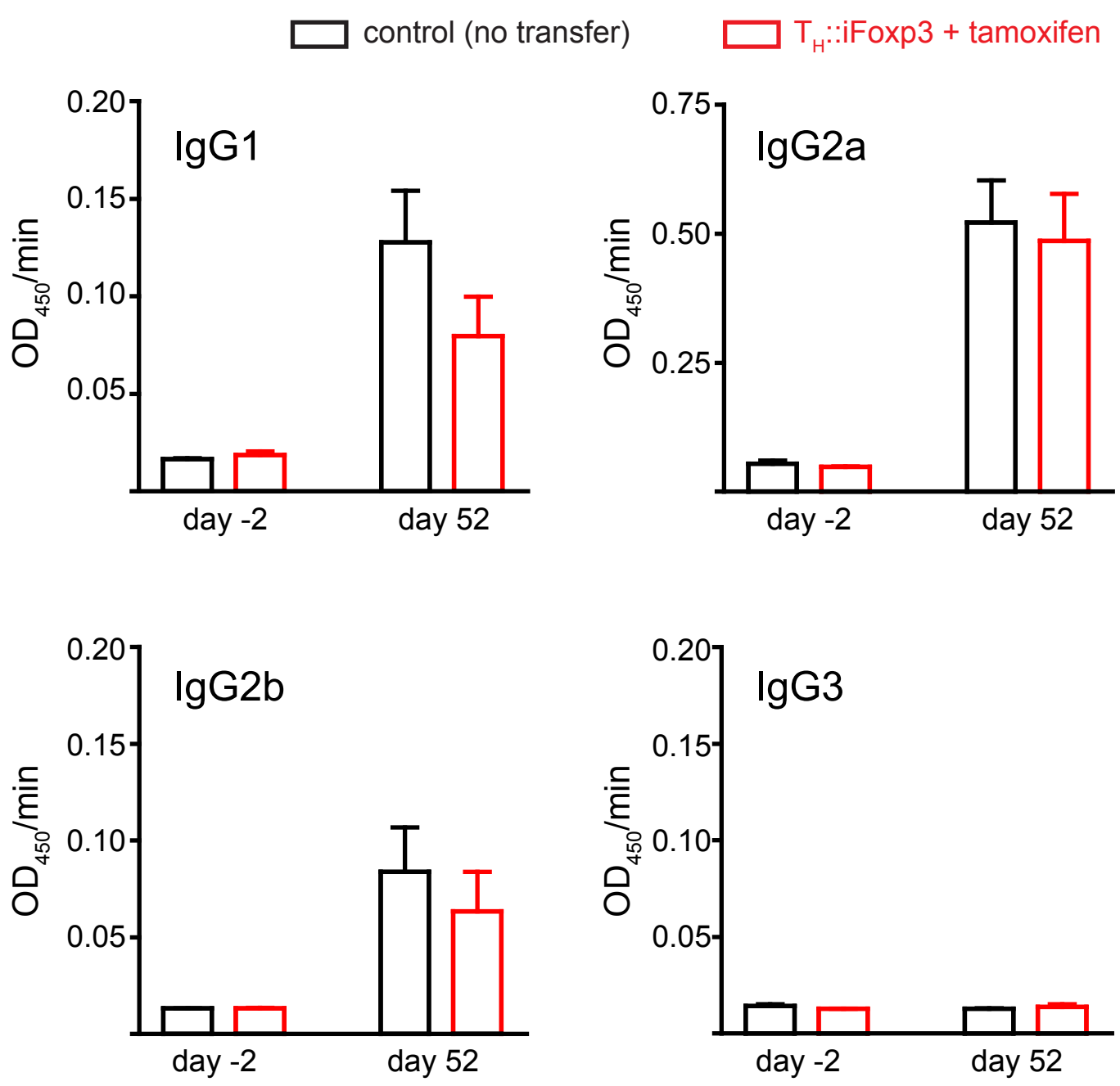
control (no transfer)
T_H::iFoxp3 + tamoxifen
IgG1
IgG2a
IgG2b
IgG3
OD_{450}/min
0.20
0.15
0.10
0.05
0.75
0.50
0.25
day -2
day 52
