# Supplementary material for: Specific Immunosuppression with Inducible Foxp3-Transduced Polyclonal T cells
Source: PLoS Biol. 2008 Nov 11;6(11):e276. doi: 10.1371/journal.pbio.0060276 (PMC2581628; doi:10.1371/journal.pbio.0060276)
Supplement: Supplementary file 6 [file pbio.0060276.sg006.pdf]

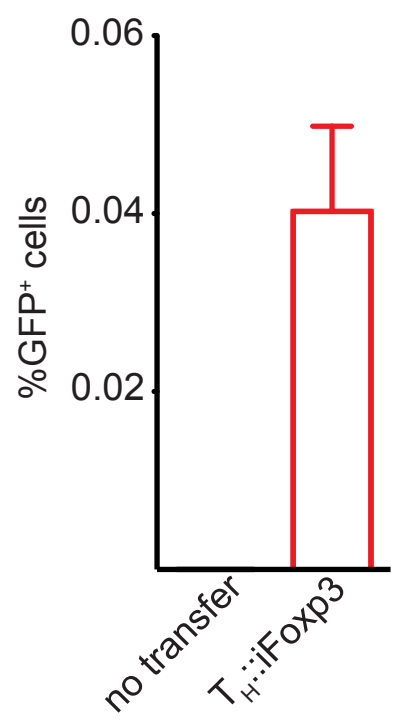
0.06
0.04
0.02
%GFP+ cells
no transfer
TH::iFoxp3
