# Supplementary material for: Specific Immunosuppression with Inducible Foxp3-Transduced Polyclonal T cells
Source: PLoS Biol. 2008 Nov 11;6(11):e276. doi: 10.1371/journal.pbio.0060276 (PMC2581628; doi:10.1371/journal.pbio.0060276)
Supplement: Supplementary file 7 [file pbio.0060276.sg007.pdf]

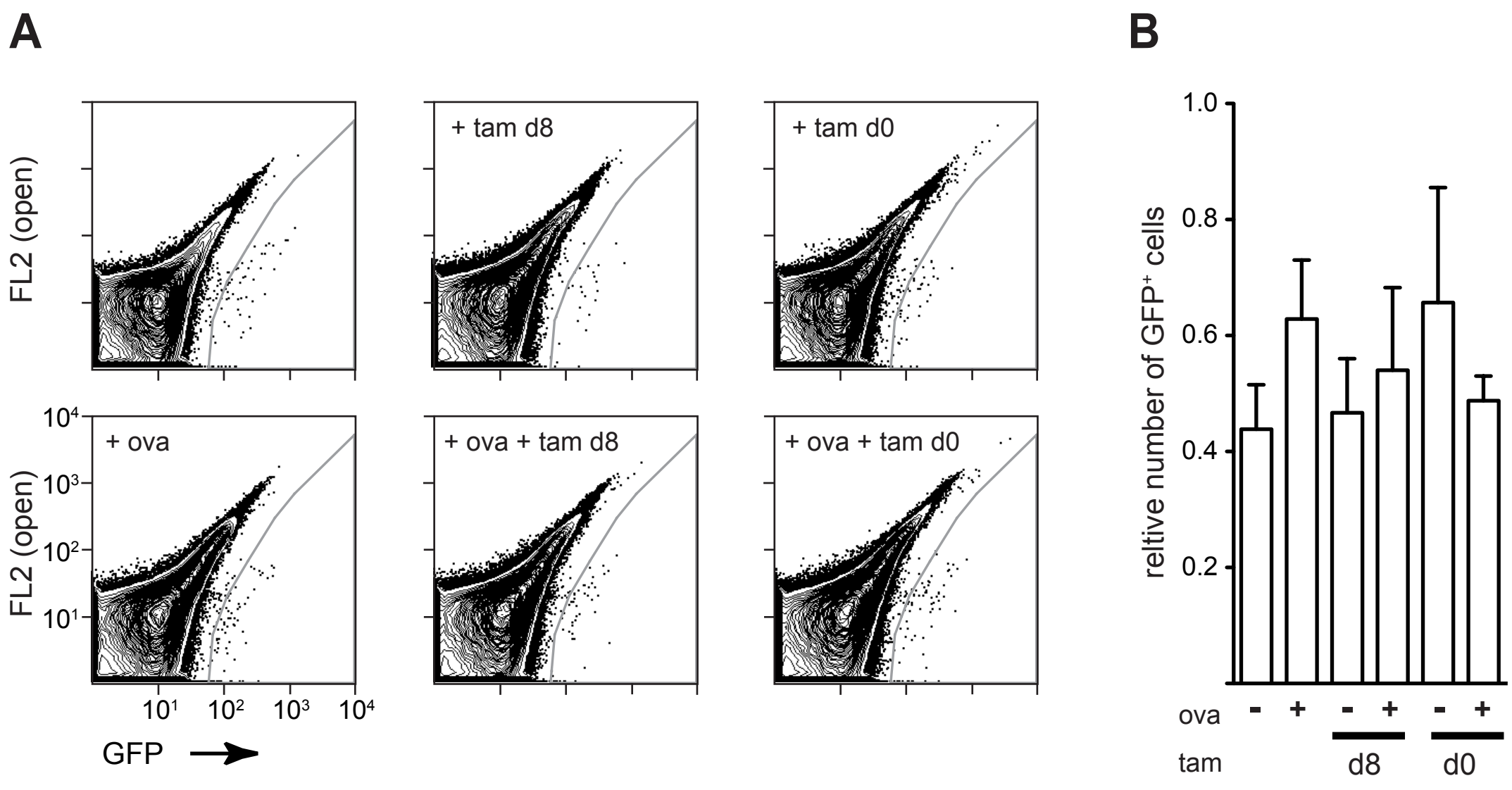
A
+ tam d8
+ tam d0
+ ova
+ ova + tam d8
+ ova + tam d0
FL2 (open)
GFP
B
reltive number of GFP+ cells
ova
tam
d8
d0
