# Supplementary material for: Specific Immunosuppression with Inducible Foxp3-Transduced Polyclonal T cells
Source: PLoS Biol. 2008 Nov 11;6(11):e276. doi: 10.1371/journal.pbio.0060276 (PMC2581628; doi:10.1371/journal.pbio.0060276)
Supplement: Supplementary file 8 [file pbio.0060276.sg008.pdf]

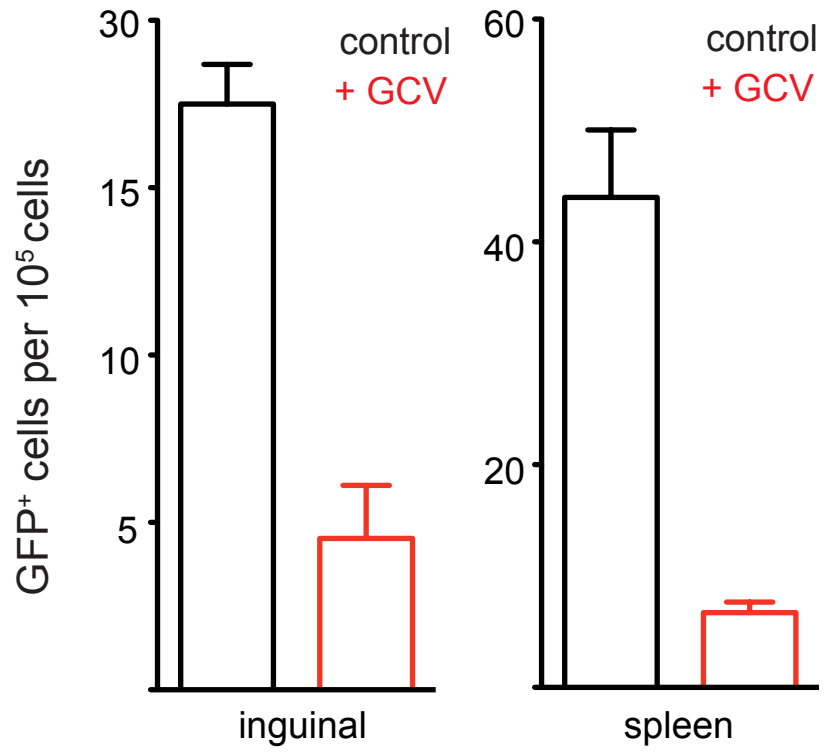

GFP+ cells per 10^5 cells
30
15
10
5
control
+ GCV
inguinal
60
40
20
control
+ GCV
spleen
